# PW01-030 – Pulmonary manifestations of FMF

**DOI:** 10.1186/1546-0096-11-S1-A83

**Published:** 2013-11-08

**Authors:** AV Sargsyan, AR Davtyan, YS Sargsyan

**Affiliations:** 1 Internal Medicine, Yerevan State Medical University, Yerevan, Armenia; 2 ArtLab Diagnostic Center, Artashat, Armenia; 3 General Medicine Student, Yerevan State Medical University, Yerevan, Armenia

## Introduction

Familial Mediterranean Fever (FMF) is an autoinflammatory disease. It is associated with vasculitis, pulmonary hemorrhage, infiltrates, and pulmonary hypertension due to amyloidosis. These complications however have been reported only rarely.

## Objectives

The aim of our study was to investigate pulmonary consequences of FMF.

## Methods

The study cohort involved 155 FMF patients (male/female 87/68). Mean age was 33,6±11,8 years in the patients group without renal amyloidosis (45 men, 35 women, n=80) and 37,8±7,4 years in the patients group with amyloidosis (42 men, 33 women, n=75). All the patients had symptoms related to the respiratory system, such as pleuritic chest pain with or without cough, dyspnea, chest tightness and frequent pneumonias. 28 patients had a history of tobacco use. Most of the patients (122) had M694V mutation, and the rest had other mutations. All the patients were on colchicine treatment at the time of the study with the exception of 2 hemodialysis patients. Laboratory tests, including CRP, SAA and capillary blood gases, ECG and chest X-ray were carried out on all the patients. 50 patients underwent Doppler echocardiography and 25 HRCT scan of the chest.

## Results

Mean C-reactive protein (CRP) and serum amyloid-A (SAA) were 17.74±13.74 mg/L vs 11,88±13.79 mg/L and 33±66.6 mg/L vs 5.25±4,45mg/L, respectively, and significantly higher in the patients group with renal amyloidosis than the mean values of the patients group without amyloidosis (P<0,000). Blood gases values (mean±SD) were within normal ranges in patients without amyloidosis, and were slightly decreased in amyloidosis patients group (PO_2_ 83.6±8.95 mm Hg, PCO_2_ 39.4±3.6 mmHg, O_2_Sat 94.6±3.38% vs. PO_2_ 74±11.36 mm Hg, PCO_2_ 35.3±4.5 mm Hg, O_2_Sat 90.1±10.26%, P<0.000). Infiltrates, ground-glass opacities, reticulonodular pattern, pleural effusion and pleural thickening, lymphadenopathy, dilatation and hypertrophy of right ventricle and increased pulmonary artery systolic pressure were more frequent findings in the patients group with amyloidosis than in the group without it, though in the group without amyloidosis they occurred as well.

## Conclusion

Our results suggest that patients with FMF and amyloidosis tend to have hypoxemia. The latter could contribute to pulmonary complications in FMF patients. On the other hand, it is possible that FMF patients without renal amyloidosis experience pulmonary manifestations and develop pulmonary complications. Respiratory symptoms in FMF patients without renal amyloidosis probably result from ongoing inflammation and early vascular alteration. Hypoxemia is a sign of advanced disease.

## Disclosure of interest

A. Sargsyan Consultant for: clinical and lab tests, A. Davtyan Consultant for: lab tests, Y. Sargsyan Consultant for: biochemical tests and statistics

